# Understanding stillbirths and maternal deaths in Pakistan: evidence from a national household survey

**DOI:** 10.7189/jogh.16.04193

**Published:** 2026-09-11

**Authors:** Sidrah Nausheen, Shabina Ariff, Muhammad Umer, Zainab Qadir, Shanila Nooruddin, Muhammad Atif Habib, Arjumand Rizvi, Ahmad Khan, Imran A Chauhadry, Henry D Kalter, Sajid Bashir Soofi, Robert E Black, Zulfiqar A Bhutta

**Affiliations:** 1 Department of Obstetrics & Gynaecology, Aga Khan University, Karachi, Pakistan; 2 Department of Paediatrics and Child Health, Aga Khan University, Karachi, Pakistan; 3 Centre of Excellence in Women and Child Health, Aga Khan University, Karachi, Pakistan; 4 Institute for Global Health & Development, Aga Khan University, Karachi, Pakistan; 5 Institute for International Programs, Bloomberg School of Public Health, Johns Hopkins University, Baltimore, Maryland, USA; 6 Centre for Global Child Health, The Hospital for Sick Children, Toronto, Ontario, Canada

**Keywords:** stillbirth, maternal mortality, verbal and social autopsy, cause-specific mortality, delays in care, Pakistan

## Abstract

**Background:**

The global impact of stillbirths and maternal deaths is enormous, with over two million deaths occurring each year, affecting public health outcomes and global development practices. Despite Pakistan’s commitment to the Sustainable Development Goals, the country faces a substantial burden of stillbirths and maternal deaths. However, data on cause-specific mortality and the timing of deaths remains limited. In this context, we aimed to estimate the cause distribution and proportion of stillbirths (antepartum and intrapartum) and maternal deaths among married women aged 12–49 years in Pakistan

**Methods:**

We conducted a cross-sectional household survey with retrospective ascertainment in urban and rural households from all four provinces of Pakistan (Sindh, Punjab, Balochistan, and Khyber Pakhtunkhwa) and three administrative regions (Gilgit Baltistan, Azad Jammu and Kashmir, and the Islamabad Capital Territory) using the sampling frame of the Pakistan National Nutrition Survey 2018. For the cause of death estimation, we calculated a target sample size of 2,100 stillbirths, allocated to provinces and regions in proportion to the population. Maternal deaths were ascertained using the same household enumeration and selection process. The mortality survey questionnaire collected information on births, stillbirths, and maternal deaths, while a Verbal and Social Autopsy questionnaire was used to assess causes of death. Following extensive training, the study teams conducted fieldwork to interview households with identified deaths. Data were captured electronically in the field to enable continuous quality control and remote monitoring. Causes of death were assigned both by trained physicians (physician-certified verbal autopsy) and by two computer-coded methods (InterVA-5 and InSilicoVA).

**Results:**

We recorded 2,076 stillbirths and 848 maternal deaths from the identified households during the 2017–2018 study period. The overall rate of stillbirth at the country level was 30.5 per 1,000 total births, while the maternal mortality ratio was 176.2 per 100,000 live births, with higher rates observed in rural areas. The leading causes of death for both stillbirths and maternal deaths were generally similar, including pregnancy-induced hypertension, obstetric haemorrhage, sepsis, and obstructed labour. Delays in seeking and accessing care, both for stillbirths and maternal deaths, resulted from issues in the identification of illness severity, decision-making, transportation issues, and delayed provision of health care once reaching a provider.

**Conclusions:**

The high stillbirth (30.5 per 1,000 births) and maternal mortality (176 per 100,000 live births) rates we observed exceed Pakistan’s national targets and the Sustainable Development Goals 2030 targets. Persistent inequities, particularly in rural Balochistan and Khyber Pakhtunkhwa, are driven by delays in recognising illness, deciding to seek care, reaching facilities, and receiving timely facility-based care. Closing these gaps will require provincial investment in emergency obstetric and newborn care, scale-up of skilled birth attendance, and community awareness of danger signs.

Stillbirths and maternal mortality are substantially impacting the health and global development of countries worldwide [1]. The World Health Organization (WHO) defines stillbirth as a loss of pregnancy or birth of a baby with no signs of life at or after 28 weeks of gestation, while maternal death refers to the death during pregnancy or within 42 days of pregnancy termination, irrespective of the cause of death (obstetric and non-obstetric/indirect) [2]. The global stillbirth rate was 13.9 per 1,000 total births in 2021, corresponding to 1.9 million deaths [3]. Analogously, the maternal mortality ratio (MMR) in 2020 was significantly higher in low-income countries, with 430 per 100,000 total births compared to 12 per 100,000 total births in high-income countries, amounting to 287,000 maternal deaths worldwide [2]. These mortality measures might be underestimated, however, because a sizable number of stillbirths and maternal deaths remain unreported [3].

The Sustainable Development Goals (SDGs) have established a target to reduce the MMR to 70 deaths per 100,000 total births or lower by 2030 [4]. Likewise, the Every Newborn Action Plan, a global movement by WHO to achieve the SDGs, aims to reduce stillbirths to not more than 12 deaths per 1,000 total births [5]. However, progress towards these goals is slower than anticipated, particularly in certain countries of South Asia and Southern Africa [6].

These outcomes remain a substantial public health challenge in Pakistan, with a maternal mortality ratio of 186 per 100,000 live births in 2018–2019 and a stillbirth rate of 30.6 per 1,000 total births in 2017–2018 [7–9]. Findings from previous studies have consistently highlighted poor maternal health, lack of provisions for antenatal care (ANC), unavailability of accessible and quality healthcare, and delayed healthcare seeking as contributing factors to this high incidence [10].

National estimates of the burden, causes, and timing of stillbirths and maternal deaths are critical for tracking progress toward the 2030 SDGs [11]. However, Pakistan lacks up-to-date cause-specific mortality data due to incomplete registration of deaths and poor certification for causes [12], leaving an urgent need for high-quality, standardised, population-based data to quantify the burden, causes, and timing of stillbirths and maternal deaths. Responding to this need, the Aga Khan University (AKU), in collaboration with Johns Hopkins University (JHU), conducted a household survey, employing new verbal and social autopsy (VASA) methods to directly estimate the rates and causes of stillbirths and maternal deaths in Pakistan.

## METHODS

We used the sampling frame of the Pakistan National Nutrition Survey (PNNS) [13], provided to the AKU by the Pakistan Bureau of Statistics. The VASA interviews were then conducted in the households where stillbirths and maternal deaths were identified in the last three preceding years. Full methodological details, including sampling, questionnaire adaptation, training, and analytical procedures, are reported in the accompanying study protocol [14].

### Households selected for mortality rates and VASA interviews

For mortality rate estimation, a census was conducted in 4,475 clusters of the sampling frame used in the PNNS [13]; 502,933 households, comprising a population of 3.4 million, were covered. The survey teams administered a mortality survey questionnaire to the population to ascertain the numbers of live births, stillbirths, and maternal deaths for the three years preceding the survey date.

We determined the target sample size for cause-of-death ascertainment by calculating the number needed to achieve 3% precision for the most common cause of stillbirth. Assuming a 50% proportion (worst case) and inflating for a design effect of 1.5 and a 15% non-response rate, we obtained 1,884, which we rounded to 2,100 stillbirths. Maternal deaths were ascertained through the same household enumeration used for stillbirths, yielding 848 deaths. This corresponds to approximately 4.1% precision for the most common cause of death, compared with the 3% design target used for stillbirths.

We then allocated the overall sample for the VASA interviews to each province in proportion to its population size. A computer-generated programme randomly selected the required number of households among those households where deaths were identified. A VASA questionnaire was completed for stillbirths (birth of a baby with no signs of life at or after seven months of gestation) and maternal deaths (death of a pregnant woman during or within 42 days of pregnancy) [2].

### Cause of death – VASA questionnaire

The VASA questionnaire we used in this study combined the WHO’s 2016 Verbal Autopsy (VA) questionnaire with the Johns Hopkins Bloomberg School of Public Health/Institute for International Programs (JHU/IIP) Social Autopsy (SA) tool [15,16]. Our adapted VASA tool has modules for assessing stillbirths and maternal deaths, including questions on the mother’s background characteristics, pregnancy, and delivery circumstances that can contribute to stillbirths and maternal deaths. The SA tool was recently updated to shorten the questionnaire; simplify and standardise its indicators, question-wording, and formatting; improve the assessment of stillbirths and maternal deaths; and examine additional preventive as well as curative factors [15]. We reviewed overlapping domains between the WHO VA and JHU SA tools and streamlined repetitive questions, while keeping the true intent of both instruments. The AKU then developed a computer-aided personal interview software, which assisted interviewers by enabling the capture of the VASA interview directly in the field on a handheld device. The VASA questionnaire was translated from English into Urdu, Pakistan’s main language, and then independently back-translated into English, where for consistency, each back-translation was compared to the original English questionnaire. The final tools were piloted in the field with VASA interviewers and a small sample of caregivers, and refined based on the observations recorded during the pilot activity.

### Data collection

#### Fieldwork and training

The VASA teams were composed of a male supervisor and female interviewers who had received seven days of extensive classroom training in the content and methodology of the VASA and two days of field training in survey implementation. Senior faculty of the AKU with prior experience conducting and implementing VASA interviews and analyses delivered five workshops as central training modules at the provincial level. The teams responsible for the cause-of-death analysis were also provided with additional training by a JHU VASA expert in a workshop on categorising causes of death using the ICD-10 [17].

The VASA interviewers were also taught to handle emotionally sensitive questions with great care and empathy. Privacy was ensured during interviews, with respondents allowed to stop or pause the discussion if they felt distressed. Interviewers were also instructed to avoid probing into or forcing discussion of traumatic details; if they noticed the respondent(s) were visibly grieved, they were to reassure them verbally and refer them to other available health workers for psychosocial support.

#### Implementation

We revisited households where the mortality survey identified a stillbirth or maternal death to conduct the VASA interview. The maximum recall period from death to VASA interview was three years. The VASA field teams worked closely with the mortality survey team, and each VASA interview was conducted soon after a death was identified. Interviewers approached the sampled households, explained the purpose of the study, identified the appropriate respondent (the main caregiver), invited them to participate, and obtained their informed consent. The interviews were structured to follow a chronological narrative, allowing respondents to naturally recount the events leading to the death with minimal recall bias. The AKU developed and managed the data control system and, during the study, managed the VASA database, including backing up data to prevent loss, transmitting data from the field to the central office, merging individual interviewers’ datasets into a single database, and monitoring for data quality.

### Data analysis

#### Cause-of-death analysis

The social autopsy analysis included data tabulation for all variables in the SA sections of the VASA questionnaire. Paediatricians, neonatologists, and obstetricians/gynaecologists who had received specific training in cause-of-death ascertainment developed the cause of maternal death and stillbirth and intrapartum/antepartum stillbirth allocation based on the narrative part of the VA questionnaire and related sections consisting of closed-ended questions on illness signs and symptoms. Two independent reviewers assessed the physician-verified verbal autopsy (PCVA) data and classified the biological causes of death. The autopsy records, including both background information and clinical data, were available to the physicians for making an accurate cause-of-death classification. In the event of disagreement between two reviewers, the case was referred to neonatologists and obstetrician-gynaecologists for adjudication.

We also used the computer-coded verbal autopsy (CCVA) InterVA-5 and InSilicoVA computer-coded analysis methods to determine the maternal causes of death [18,19]. Causes were assigned using PCVA and by CCVA using InterVA-5 and InSilicoVA, which differ in assumptions, priors, and handling of symptom patterns (deterministic physician judgment *vs*. probabilistic modelling). To compare PCVA with CCVA, we calculated percent agreement and Cohen’s kappa for the three leading causes and the absolute difference in cause-specific mortality fractions. Both the PCVA and the CCVA were applied to all cases in parallel. InterVA-5-Bayesian model and InSilicoVA – probabilistic latent-class model were used to assess the cause of death [19,20]. The CCVA was used to compare and validate cause-of-death distributions with the physician-assigned causes. In cases of disagreement between methods, the final cause of death recorded was based on the physician's consensus diagnosis following arbitration.

We derived the delay variables from the structure questions, which were part of the VASA tool, adapted from the JHU/IIP SA instrument. We coded each of the delays (recognising danger signs, making the decision, transportation, and receiving care) in binary variables (yes/no) and recorded them accordingly based on the respondents’ answers. Here, we distinguished a delay in recognition of severity or danger signs; a delay in decision to seek care; a delay in transportation to facility; and a delay in the provision of care at the facility.

#### Statistical analysis

We incorporated the survey design (primary sampling unit identifiers, household clustering, and sampling weights) in our analysis to ensure that variance estimates and 95% confidence intervals (CIs) appropriately accounted for the clustering effect. All analyses were conducted in Stata, version 18.0 (StataCorp LLC, College Station, Texas, USA) using the ‘svy’ commands/survey package to incorporate stratification, primary sampling unit identifiers, and sampling weights. We reported point estimates with 95% CIs and assessed differences in cause-specific mortality fractions across PCVA and CCVA using percent agreement and Cohen’s kappa. The reported mortality estimates were calculated using aggregated counts of births and deaths identified through the line-listing process.

## RESULTS

A total of 2,912 (99%) interviews were completed among 2,924 households sampled for stillbirths and maternal deaths, and the final sample included 2,076 stillbirths and 848 maternal deaths. For stillbirths, the mean recall period was 1.04 (95% confidence interval (CI) = 0.99–1.08) years, which was significantly different from the mean recall period of 1.13 (95% CI = 1.05–1.19) years for maternal deaths.

**Figure 1:**
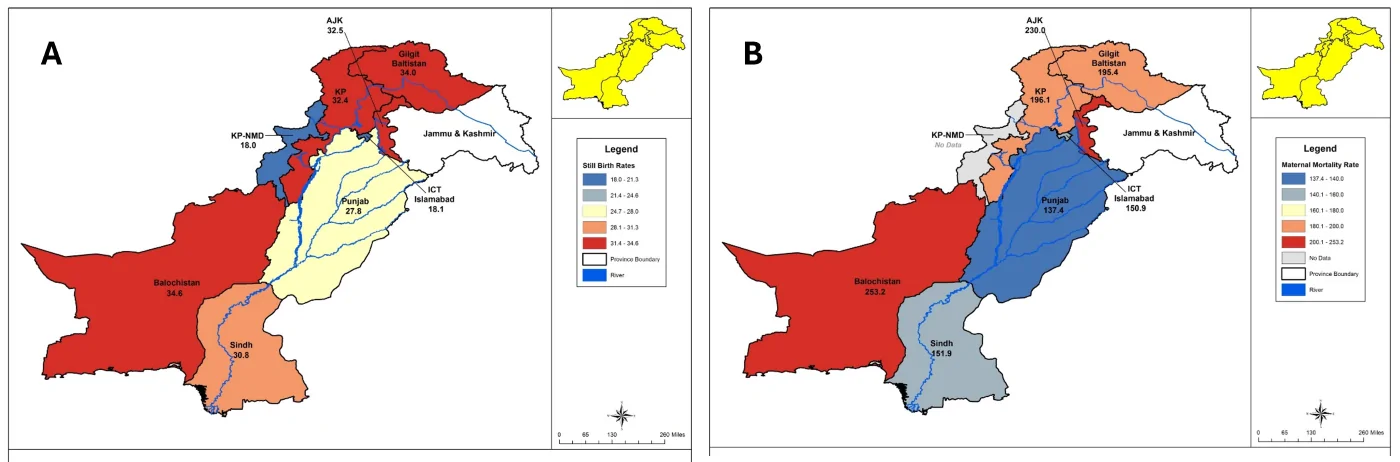
Distribution of maternal mortality (**Panel A**) and stillbirths (**Panel B**) across provinces and administrative regions in Pakistan. AJK – Azad Jammu Kashmir, ICT – Islamabad Capital Territory, KP – Khyber Pakhtunkhwa, KP-NMD – Khyber Pakhtunkhwa Newly Merged Districts.

The MMR for the 2017–2018 period was 176.2 per 100,000 live births (95% CI = 163.0–189.2), with higher rates in rural (178.4; 95% CI = 162.6–194.1) compared to urban areas (170.5; 95% CI = 146.8–193.9). The stillbirth rate was 30.5 per 1,000 total births (95% CI = 30.1–31.0), with a slightly higher rate in rural (95% CI = 31.0; 30.5–31.5) compared to urban areas (29.4; 95% CI = 28.7–30.2). Balochistan had the highest stillbirth rate (34.6; 95% CI = 33.5–35.7), while Punjab had the lowest (27.8; 95% CI = 27.2–28.4) (**Figure 1**, Panels A and B, **Table 1**).

**Table 1:** Mortality rates

	Stillbirth rate, x̄ (95% CI)	Maternal mortality ratio, x̄ (95% CI)
**National**	30.5 (30.1–31.0)	176.2 (163.0–189.2)
Rural	31.0 (30.5–31.5)	178.4 (162.6–194.1)
Urban	29.4 (28.7–30.2)	170.5 (146.8–193.9)
**Province**		
Punjab	27.8 (27.2–28.4)	137.4 (118.7–156.0)
Sindh	30.8 (29.8–31.9)	151.9 (119.1–184.0)
KP	32.4 (31.2–33.5)	196.1 (160.4–231.1)
Balochistan	34.6 (33.5–35.7)	253.2 (219.3–286.4)
KP-NMD	18.0 (14.0–21.7)	*
ICT	18.1 (15.1–20.9)	150.9 (19.2–275.4)
GB	34.0 (31.9–36.0)	195.4 (136.5–252.5)
AJK	32.5 (29.8–35.1)	230.0 (137.5–318.7)

AJK – Azad Jammu Kashmir, GB – Gilgit Baltistan, ICT – Islamabad Capital Territory, KP-NMD – Khyber Pakhtunkhwa Newly Merged Districts, x̄ – mean

*Estimate not reported because the sample size was insufficient to generate a reliable mortality estimate.

### Background characteristics of stillbirths

The respondents were primarily mothers (90.1%) and grandmothers (3.4%). The average household density was 3.8 (standard deviation (SD) = 2.0) persons per room, while the mean age at the current residence was 17.0 (SD = 16.6) years. Improved sources of water and sanitation were accessible to more than 90% of the inhabitants, while nearly 54% of stillbirth cases were able to reach the nearest health facility within 0–0.5 hours. In terms of place of delivery, 1,496 (72.4%) of the stillbirths occurred in health facilities, while 553 (26.8%) occurred at home. The pathway delays in stillbirth cases included delays in recognizing the severity of maternal illness and danger signs (n = 1,789, 88%), decision-making (n = 853, 42%), transportation issues (n = 293, 14%), and receiving appropriate services from providers (n = 896, 44%) (Table S1 in the **Online Supplementary Document**).

### Causes of stillbirths

Of the 2,076 stillbirths, 1,369 (65.9%) occurred during the antepartum period (foetal death before the onset of labour) and 707 (34.1%) during the intrapartum phase (foetal death during labour and before delivery), characterised based on foetal movement, absence or presence of foetal heart sounds, and skin appearance (maceration/fresh stillbirth) [21]. There was a male-female ratio of 1.63 for intrapartum stillbirths. Pregnancy-induced hypertension was the most frequent cause of stillbirths (n = 688, 33%), followed by maternal infections (n = 256, 12%), antepartum haemorrhage (n = 230, 11%), and obstructed labour (n = 217, 10%) (**Table 2**).

**Table 2:** Stillbirth causes of death per the PCVA

	Intrapartum		Antepartum	
**Cause of death**	**n (%)**	**% of causes**	**n (%)**	**% of causes**
Pregnancy-induced hypertension	222 (31.4)	32.3	466 (34.0)	67.7
Maternal infections that can affect the foetus	83 (11.7)	32.4	173 (12.6)	67.6
Antepartum haemorrhage	83 (11.7)	36.1	147 (10.7)	63.9
Obstructed labour	95 (13.4)	43.8	122 (8.9)	56.2
Other obstetric complications	38 (5.4)	42.7	51 (3.7)	57.3
Congenital malformations	10 (1.4)	13.9	62 (4.5)	86.1
Other specific perinatal causes	20 (2.8)	41.7	28 (2.0)	58.3
Maternal accident/injury	9 (1.3)	39.1	14 (1.0)	60.9
Maternal medical conditions	1 (0.1)	16.7	5 (0.4)	83.3
Gestational diabetes	2 (0.3)	40.0	3 (0.2)	60
Undetermined	144 (20.4)	32.6	298 (21.8)	67.4
Total	707 (100)	34.1	1,369 (100)	65.9

### Background characteristics of maternal deaths

The respondents for maternal deaths were most often in-laws (37.4%), followed by sisters and mothers (21.7% and 15.9%). Regarding household characteristics, the average household density was 3.8 (SD = 2.0) persons per room, while the average time at the current residence was 20.6 (SD = 17.5) years. Moreover, 92.3% of residents had access to improved water sources, while approximately 71% had access to improved sanitation facilities. Nearly 45% of maternal mortality cases were able to reach the nearest health facility within 0–0.5 hours. In terms of place of maternal deaths, the most frequent settings where these events occurred were healthcare facilities (n = 490, 58.2%), at homes (n = 222, 26.4%), and while traveling to a healthcare provider or facility (n = 105, 12.5%). The pathway delays in maternal death cases included delays in recognising the severity of illness and danger signs (n = 709, 84%), decision-making (n = 302, 36%), transportation issues (n = 151, 18%), and receiving appropriate services from providers (n = 563, 67%) (Table S2 in the **Online Supplementary Document**).

### Causes of maternal deaths

The analysis conducted using InterVA5 and InSilicoVA found that direct maternal causes such as obstetric haemorrhage, pregnancy-related sepsis, and pregnancy-induced hypertension were responsible for 85.8% of maternal deaths. In contrast, other causes such as non-communicable diseases, accidents, pneumonia, infectious diseases, anaemia, and non-maternal causes accounted for the remaining maternal deaths. In contrast, when using the PCVA method, 66.6% of maternal deaths occurred because of direct maternal causes, while 25% were attributable to other indirect causes. There were differences in the levels of individual causes identified by the CCVA and PCVA methods. However, both methods identified the exact three leading causes, with obstetric haemorrhage being the most common cause, followed by pregnancy-induced hypertension and maternal sepsis (**Table 3**).

**Table 3:** Maternal causes of death*

	InterVA5	InSilicoVA	Average %	PCVA
**Direct maternal causes**	725 (85.5)	730 (86.1)	85.80	565 (66.6)
Obstetric haemorrhage	477 (56.3)	366 (43.2)	49.70	262 (30.9)
Pregnancy-induced hypertension	125 (14.7)	98 (11.6)	13.10	200 (23.6)
Pregnancy-related sepsis	98 (11.6)	201 (23.7)	17.60	52 (6.1)
Abortion-related death	9 (1.1)	10 (1.2)	1.10	13 (1.5)
Anaemia of pregnancy	5 (0.6)	19 (2.2)	1.40	0 (0)
Other specified and unspecified maternal causes of death	5 (0.6)	5 (0.6)	0.60	17 (2.0)
Ruptured uterus	5 (0.6)	24 (2.8)	1.70	5 (0.6)
Ectopic pregnancy	1 (0.1)	5 (0.6)	0.40	3 (0.4)
Obstructed labour	0 (0)	2 (0.2)	0.10	13 (1.5)
**Indirect maternal causes**	76 (9.0)	59 (7.0)	8.00	212 (25)
Other non-communicable diseases	52 (6.1)	36 (4.2)	5.20	1 (0.1)
Pneumonia	7 (0.8)	8 (0.9)	0.90	0 (0)
Cause not possible to determine from verbal autopsy	5 (0.6)	0 (0)	0.30	52 (6.1)
HIV/AIDS-related death	5 (0.6)	4 (0.5)	0.50	0 (0)
Tuberculosis	5 (0.6)	5 (0.6)	0.60	2 (0.2)
Cancers and neoplasms	1 (0.1)	0 (0)	0.10	0 (0)
Diarrheal diseases	1 (0.1)	1 (0.1)	0.10	0 (0)
Infectious diseases	0 (0)	5 (0.6)	0.30	1 (0.1)
Other specific non-maternal causes	0 (0)	0 (0)	0.00	79 (9.3)
Pre-existing medical conditions exacerbated by pregnancy	0 (0)	0 (0)	0.00	60 (7.1)
Severe anaemia	0 (0)	0 (0)	0.00	17 (2)
**Coincidental causes**				
Accidents and injuries (including self-harm)	47 (5.5)	59 (7)	6.30	62 (7.3)
Missing	0 (0)	0 (0)	0.00	9 (1.1)

PCVA – physician-certified verbal autopsy

*Presented as n (%) unless specified otherwise.

Provincial disparities likely reflect differences in facility readiness and effective coverage of emergency obstetric and newborn care. Although 58.2% of maternal deaths occurred in facilities and >70% of stillbirths occurred after facility delivery, reported delays in receiving appropriate services were common (maternal deaths: 66.6%; stillbirths: 44.0%), indicating gaps in triage, timely treatment (*e.g.* MgSO_4_, antibiotics, blood), and referral within facilities. These deficits were more frequently reported in Balochistan and Khyber Pakhtunkhwa relative to Punjab and Sindh, aligning with their higher mortality and predominantly rural populations.

**Figure 2:**
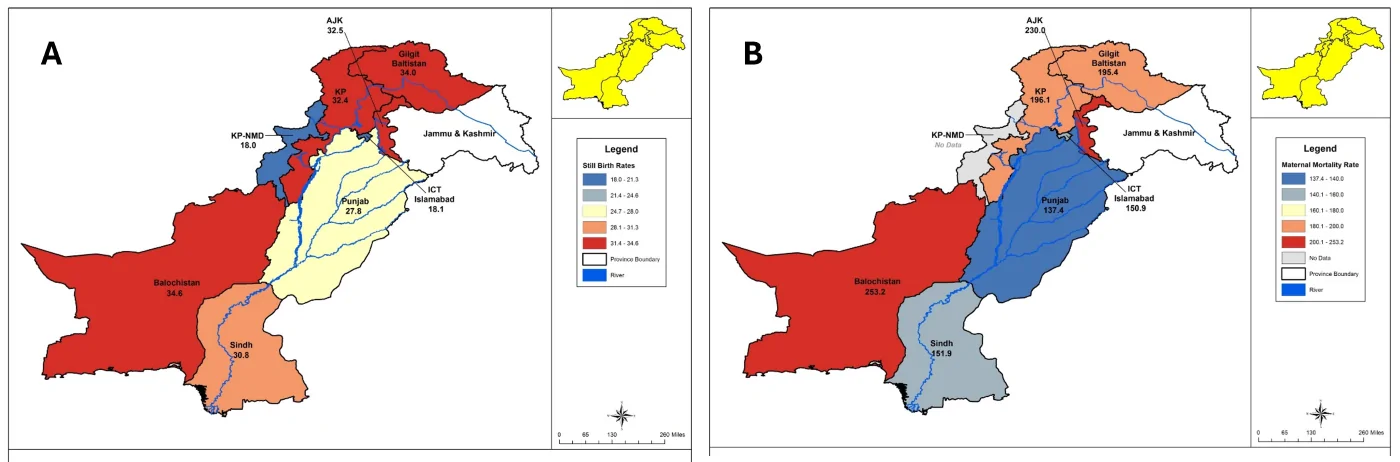
Urban–rural differences in maternal mortality per 100,000 live births (**Panel A**) and stillbirths per 1,000 births (**Panel B**) in Pakistan.

Urban-rural gradients were evident across provinces (**Figure 2**, Panels A and B). In aggregate, rural settings exceeded urban for both stillbirths and maternal mortality; this gap was most pronounced in Balochistan and Khyber Pakhtunkhwa, consistent with the national pattern (**Table 1**).

We identified the same top three direct causes (obstetric haemorrhage, pregnancy-induced hypertension, and sepsis) across methods, though their rank order and cause-specific mortality fractions differed (**Table 3**). Agreement between PCVA and the automated verbal autopsy methods varied by cause of death but was generally low. For obstetric haemorrhage, the highest level of agreement was observed, with agreement percentages of 37.23% between PCVA and InterVA-5 and 33.21% between PCVA and InSilicoVA. However, the corresponding kappa coefficients were very low, at 0.0307 and 0.0489, respectively.

For pregnancy-induced hypertension, agreement between PCVA and CCVA was low, at 23.22% and 18.73%, and with kappa values of 0.0115 and 0.0176 for InterVA-5 and InSilicoVA, respectively. Similarly, agreement for pregnancy-related sepsis was poor, at 8.77% and 11.40%, respectively, with corresponding kappa coefficients of 0.0337 and 0.0294.

Overall, the findings indicate varying levels of concordance PCVA and CCVA approaches in the assignment of specific maternal causes of death. The highest agreement was observed for obstetric haemorrhage, while lower levels of agreement were observed for pregnancy-induced hypertension and pregnancy-related sepsis.

## DISCUSSION

In this study, we employed the VASA method, which identifies signs and symptoms through a structured interview with the primary caregiver. Our findings highlight that the stillbirth rate in Pakistan was 30.5 deaths per 1,000 total births, while the MMR was 176.2 per 100,000 births during 2017–2018. These statistics represent no substantial improvement in comparison with the previously reported 30.6 stillbirths per 1,000 births (2017–2018) and MMR of 186 per 100,000 births (2018–2019) [7]. Similarly, the United Nations Inter-agency Group for Child Mortality Estimation reported a stillbirth rate of 29.38 per 1,000 total births in 2020 [22]. Estimates from the UN Maternal Mortality Estimation Inter-Agency Group indicate that Pakistan’s MMR declined from 368 per 100,000 live births in 1985 to 154 per 100,000 births in 2020, a reduction of 214 per 100,000 [2]. We observed higher mortalities for both stillbirths and maternal deaths in KP and Balochistan, in contrast to Punjab and Sindh, particularly in rural settings, as opposed to urban areas. The findings are consistent with the national estimates reporting 26% higher MMR in rural (199 per 100,000 births) than urban areas (158 per 100,000 births) in 2018–2019 [6]. Additionally, a survey conducted in 2017–2018 indicated a 65% higher prevalence of stillbirths in rural (184 per 1,000 total births) compared to urban settings (65 per 1,000 total births) [7,23], emphasising the major challenges in healthcare access and geographical barriers in such contexts [24–26].

Compared with Every Newborn Action Plan target (12 stillbirths per 1,000 births by 2030), the stillbirth rates we observed here and which were identified in the Pakistan Demographic and Health Survey (PDHS) 2017–2018 are significantly higher [4,27]. Evidence shows that 8.6 stillbirths occur for every 200 births in Pakistan, in contrast to one stillbirth in high-income countries [27]. Regarding background and maternal characteristics, most stillbirths occurred in women of advanced maternal age, with lower educational backgrounds, in poorer wealth quintiles, with complicated pregnancies, and who had not received formal pregnancy care in the last trimester. Most women who had a stillbirth were delivered by skilled birth attendants (90%); however, only 39.3% of women made four or more ANC visits. Most of the mothers reached health facilities within one hour, and a quarter of stillbirths occurred at home, indicating inadequate access to and utilisation of healthcare. These determinants are consistent with the contributing factors to stillbirth identified from low- and middle-income countries, including a maternal age >30 years, poor socioeconomic and educational backgrounds, and poor ANC [28,29]. We observed a male preponderance among intrapartum stillbirths, with a male-to-female ratio of 1.63, which is consistent with the global pattern of higher male foetal vulnerability. These differences in cause-specific mortality fractions across analytic methods are also consistent with the literature, where the risk of stillbirth remained higher among males as compared to females, as high testosterone levels suppress the immune system [30,31].

Regarding causes of death, pregnancy-induced hypertension/pre-eclampsia/eclampsia, maternal infections, antepartum haemorrhage, and obstructed labour were reported as the primary causes according to PCVA for both antepartum and intrapartum cases. In Pakistan, national ANC guidelines recommend routine blood pressure measurement and urine protein testing at each visit to screen for pre-eclampsia and eclampsia. However, the implementation of these practices remains inconsistent, especially in rural and resource-limited settings. Shortages of trained providers, inadequate equipment, and limited adherence to ANC protocols often lead to missed opportunities for early detection and timely management of hypertensive disorders during pregnancy, which may contribute to the high proportion of stillbirths related to pregnancy-induced hypertension we observed here.

Literature from developing countries also reported that conditions associated with stillbirths included maternal morbidities (diabetes, HIV, syphilis), foetal causes (congenital anomalies, infections), placental abruptions, intrapartum asphyxia, umbilical cord prolapse, chorioamnionitis, uterine rupture, and unexplained causes, among others [29]. A study conducted in India using hospital assessment and VA determined that the frequent causes of stillbirth included pregnancy-induced hypertension (30%), antepartum haemorrhage (16%), underlying maternal illness (12%), congenital malformations (12%), and obstetric complications (10%) [32]. Results from the largest prospective study conducted in South Asia on the causes of stillbirth, the Project to Understand and Research Preterm Pregnancy Outcomes and Stillbirths, focused on foetal causes of death, including intrauterine hypoxia, foetal infections, and growth restrictions, where hypertension was the primary maternal cause of death, followed by anaemia and maternal infections [33]. Both these studies’ results support the VASA’s finding of pregnancy-induced hypertension, causing a third of all stillbirths and two-thirds of the stillbirths occurring antepartum.

The MMR reported in this study (176 per 100,000 live births) is also substantially higher than in other developing countries, such as South Africa (128 per 100,000 live births), Bangladesh (168 per 100,000 live births), and India (107 per 100,000 live births) and far exceeds the SDG 2030 target of 70 per 100,000 live births [4,34]. Nearly half of the deaths occurred among 20–29-year-olds, mostly in health facilities. In addition, MMR was predominantly among women from poor wealth quintiles and with no educational background. Reports from the PDHS and the United Nations Children’s Fund also emphasised poor educational status being a key factor in poor maternal outcomes, resulting from limited access to health care [12,35,36]. The causes of maternal deaths we identified here included direct, indirect, and coincidental causes. The most common direct maternal causes identified from both methods (PCVA and InterVA5/InSilicoVA) were obstetric haemorrhage, pregnancy-induced hypertension, and sepsis. However, there are variations observed in both methods attributed to methodological disparities, encompassing subjectivity (PCVA) *vs*. automation (CCVA), data quality, reporting practices, and regional healthcare practices [37,38]. These findings are aligned with previous data from the PDHS 2006–2007 and the Pakistan Maternal Mortality Survey 2019 [7,8]. Evidence from national and international literature also indicates higher maternal mortality risks in cases of obstructed labour, antepartum haemorrhage, and hypertensive disorders (pre-eclampsia/eclampsia) [25,39–41]. Other indirect causes included infectious diseases (pneumonia, tuberculosis, HIV/AIDS, and/or anaemia).

More than half of maternal deaths in our study (58.2%, *i.e.* 3 in 5) and a significant portion of stillbirths (72.4%) occurred within health facilities, which is indicative of delays in healthcare pathways. Specifically, delays in decision-making, transportation or logistical issues, and shortages of healthcare services from providers were the most prevalent factors contributing to mortality. The findings are consistent with previous research which showed significant association between delays in healthcare access and adverse pregnancy outcomes, including stillbirths and maternal deaths [42].

While the WHO proposes a range of evidence-based interventions against preventable maternal mortality and stillbirths, their coverage in Pakistan remains inadequate [5]. The absence of adequate financial and human resources, insufficient political commitment, and weaknesses in health infrastructure create significant obstacles to achieving the health-related SDGs by 2030 [43]. In addition, the inequalities outlined in the PDHS between urban and rural access and utilisation of health services pose a substantial challenge in improving the maternal, neonatal, and child health indicators. Prioritising recognition and early care-seeking symptoms of the most common pregnancy and labour/delivery complications at both the health facility and community level could help with preventing complications during pregnancy, the occurrence of stillbirths, and maternal deaths in the delivery phase. Some strategies to consider include expanding service delivery channels, raising awareness among women and their spouses about the significance of ANC and recognising warning signs, providing regular training for health care providers, educating community midwives and traditional birth attendants to identify and refer women with complications, improving access to skilled birth attendance, strengthening the referral system, and establishing community funds to support those requiring referrals in critical situations [43,44].

### Strengths and limitations

The major strength of our study is that the data were collected across Pakistan to obtain a generalisable, representative sample. To minimise language and cultural barriers, trained staff were recruited from the local population, following their culture and norms. Real-time data were collected electronically to eliminate errors and falsification, while GPS coordinates of each cluster were recorded to support spatial analysis and quality assurance.

In our study, CCVA methods could be validated only against PCVA and clinical records, not against full diagnostic autopsy (the reference standard for cause-of-death estimation [19,20]). Our cause-of-death estimates differed between PCVA and CCVA, particularly in the proportional attribution of haemorrhage versus sepsis. We further encountered challenges related to inadequate health-seeking behaviour resulting in most deaths not being registered at the government level and reliance on average recall periods of about one year. Recall bias from respondents and the emotional nature of the interviews may have also affected some responses and led to the recall bias which could lead to both under- and over-reporting of symptoms particularly maternal deaths. Our VASA interviews faced limitations in linking symptoms to specific underlying diagnoses, particularly for rare or co-occurring causes and providing psychological support to family members/respondents during the recall of stillbirth and maternal mortality events. However, the teams counselled these individuals to visit nearby health facilities in cases of emergencies.

### Policy and programme implications

Our findings of higher stillbirth and maternal mortality rates in rural areas and in Balochistan and Khyber Pakhtunkhwa; the concentration of events within facilities, and delays in recognition, referral, and receipt of appropriate care indicate five immediate priorities. First, urban-rural and provincial gaps must be narrowed by prioritising Balochistan and Khyber Pakhtunkhwa for funding, staffing, and supervision, while expanding skilled ANC, intrapartum monitoring, and timely referral through mobile outreach and task-sharing. Second, facility readiness and quality of emergency newborn and obstetric care need to be strengthened with 24/7 availability of signal functions, routine use MgSO_4_ for pre-eclampsia/eclampsia, active management of the third stage of labour, prophylactic/therapeutic antibiotics, point-of-care haemoglobin testing, and blood availability through district blood hubs with rapid crossmatch and transport. Third, referral and transportation systems need to be established through provincial referral coordination centres, GPS-enabled ambulance dispatch, and community emergency transport funds at the union council level. Fourth, ANC quality needs to be improved by scaling quality bundles, including blood pressure, urine, and blood testing, danger-sign counselling, and clear escalation protocols, supported by routine audits and feedback. Finally, maternal and perinatal death surveillance and response need to be institutionalised through no-blame audits at secondary and tertiary facilities, with findings fed into provincial quality forums. Simultaneously, data systems need to be strengthened by aligning definitions with WHO standards, reinforcing civil registration and vital statistics systems, and ensuring routine verbal autopsy where certification is weak.

## CONCLUSIONS

Our national VASA study estimated a stillbirth rate of 30.5 per 1,000 total births and a MMR of 176 per 100,000 live births in Pakistan during 2017–2018 – both well above national and global targets – and identified persistent gaps in the provision of timely emergency care with higher prevalence in rural settings, underscoring the substantial burden of adverse pregnancy/maternal and neonatal health outcomes. Stillbirths and maternal deaths shared a similar set of leading direct causes – hypertensive disorders, obstetric haemorrhage, sepsis, and obstructed labour. We also observed significant pathway delays in the recognition of the severity of conditions, decision-making, transportation issues, and receiving care from providers. Our findings indicate a critical need for targeted interventions to address health system inequities, as well as a need for continued collaborative efforts between the federal government, provincial health departments, donors, professional bodies, and community organisations and strengthening of healthcare service delivery, health promotion, and prevention efforts to reduce the burden of preventable healthcare issues among pregnant women, thereby improving the maternal and neonatal health outcomes in Pakistan.

## Additional material


Online Supplementary Document

## Data Availability

**Data availability:** The data will be made available on request to the corresponding author
