# Supplementary material for: Understanding stillbirths and maternal deaths in Pakistan: evidence from a national household survey
Source: J Glob Health. 2026 Sep 11;16:04193. doi: 10.7189/jogh.16.04193 (PMC13564933; doi:10.7189/jogh.16.04193)
Supplement: Supplementary file 1 [file jogh-16-04193-s001.pdf]

Supplement to: Nausheen S, Arif S, Umer M, Qadir Z, Nooruddin S, Habib MA, Rizvi A, Khan A, Chauhadry IA, Kalder HD, Soofi SB, Black RE, Bhutta ZA. Rates and causes of neonatal and under-five mortality: insights from a national household survey in Pakistan. J Glob Health. 2026;16:04211.

Table S1. Maternal and ANC care seeking characteristics for 2070 stillbirths*

**Maternal age in years**	
<20	113 (5.5)
20–24	441 (21.3)
25–29	605 (29.2)
≥30	891 (43.0)
Does not know	20 (1.0)
**Mean age in years, x̄ (SD)**	28.5 (6.3)
**Maternal education**	
None	1364 (65.9)
Primary	252 (12.2)
Secondary	321 (15.5)
Above secondary	132 (6.4)
Does not know	1 (0.0)
**Years of schooling (n = 701), x̄ (SD)**	8.3 (3.4)
**Age when first married in years (n = 1994), x̄ (SD)**	20.2 (4.3)
**Prior births, including stillbirths**	
No birth	355 (17.1)
1–3 births	1062 (51.3)
≥4	608 (29.4)
Refused/does not know	45 (2.2)
**ANC visits**	
None	502 (24.3)
1	153 (7.4)
2	285 (13.8)
3	317 (15.3)
≥4	813 (39.3)
**Skilled ANC provider**	1548 (97.4)
**ANC components**	
Blood pressure measured	1336 (84.0)
Urine sample collected	1064 (66.9)
Blood sample collected	985 (61.9)
Provided advice on eating high-energy and high-protein foods	906 (57.0)
Provided knowledge about the danger signs during pregnancy	445 (28.0)
Provided knowledge of where to go if had any danger signs	414 (26.0)
**Quality ANC (all six components)**	249 (15.7)
**Knowledge of danger signs during pregnancy**	
Vaginal bleeding	401 (19.4)
Convulsions/fits	193 (9.3)

Severe headache with blurred vision	287 (13.9)
Fever and too weak to get out of bed	210 (10.1)
Severe abdominal pain	297 (14.3)
Fast or difficult breathing	131 (6.3)
Painful contractions every 20 min or less for 12 h or more	168 (8.1)
Broken water for 12 h or more	232 (11.2)
Bloody, sticky discharge for 12 h or more	134 (6.5)
No immediate danger signs mentioned	1165 (56.3)
**Tetanus-protected – at least two shots in current or one shot in current and at least one in previous**	841 (40.6)
**Complications in pregnancy in the last three months**	702 (33.9)
**Complications†**	
Maternal sepsis	126 (17.9)
Eclampsia/pre-eclampsia	306 (43.6)
Maternal anaemia	186 (26.5)
Maternal haemorrhage	259 (36.9)
Water broke six hours or more before labour	167 (23.8)
Diabetes	30 (4.3)
**Formal care seeking for pregnancy complications in the last three months**	
Yes	256 (36.5)
No	446 (63.5)
**Delivery place**	
Home	553 (26.8)
Health facility	1496 (72.4)
Route to HF	16 (0.8)
**Skilled birth attendant**	1869 (90.3)
**Labour and delivery duration in hours**	
<1	233 (11.3)
1–4	995 (48.1)
5–8	383 (18.5)
9–12	167 (8.1)
>12	268 (12.9)
Does not know	24 (1.2)
**Caesarean section deliveries**	395 (19.1)
**Intrapartum**	707 (100.0)
Length of pregnancy before the child was born in months, $\bar{x}$ (SD)	8.7 (0.8)
Women having at least one complication before labour began	203 (28.7)
Pregnancy complications before labour began	
Maternal sepsis	40 (5.7)
Eclampsia/pre-eclampsia	81 (11.5)
Maternal anaemia	57 (8.1)
Maternal haemorrhage	71 (10.0)
Water broke six hours or more before labour	47 (6.6)
Diabetes	9 (1.3)
Women having any complications during labour†	170 (24.0)
Pregnancy complications during labour†	
Maternal sepsis	53 (7.5)

Eclampsia/pre-eclampsia	73 (10.3)
Maternal anaemia	48 (6.8)
Maternal haemorrhage	80 (11.3)
**Antepartum**	1369 (100.0)
Length of pregnancy before the child was born in months, x̄ (SD)	8.1 (1.2)
Women having at least one complication before labour began	502 (36.7)
Pregnancy complications before labour began†	
Maternal sepsis	88 (6.4)
Eclampsia/pre-eclampsia	227 (16.6)
Maternal anaemia	129 (9.4)
Maternal haemorrhage	189 (13.8)
Water broke six hours or more before labour	122 (8.9)
Diabetes	22 (1.6)
Women having any complications during labour†	418 (30.5)
Pregnancy complications during labour	
Maternal sepsis	102 (7.5)
Eclampsia/pre-eclampsia	194 (14.2)
Maternal anaemia	131 (9.6)
Maternal haemorrhage	221 (16.1)
**Delays for stillbirths and maternal deaths**	
**Delay in recognising the severity of illness and danger signs (n = 2035)**	1789 (87.9)
**Delay in decision-making (n = 2035)**	853 (41.9)
No male member/any member available to accompany	94 (4.6)
No available person to take care of the children, the home, and the livestock	129 (6.3)
Fear of being ill-treated in the health facility	116 (5.7)
Lack of encouragement from relatives and community members to seek care	294 (14.4)
Lack of money to pay for medical expenses and cost of transportation	573 (28.2)
Too sick to travel	13 (0.6)
Fear of long waiting time	0 (0.0)
**Delay due to transport (n = 2035)**	293 (14.4)
Distance from a woman's home to a facility or provider	156 (7.7)
Lack of roads or poor condition of roads	33 (1.6)
Lack of emergency transportation	210 (10.3)
**Delay in getting appropriate service from the provider (n = 2035)**	896 (44.0)
Lack of health care personnel care provider	491 (24.1)
Gender-related issues between patients and health care providers	16 (0.8)
Shortages of supplies, *i.e.* emergency medicines or blood	10 (0.5)
Lack of competence of health care providers to provide emergency care	563 (27.7)
Delayed referral	170 (8.4)
Long waiting time	0 (0.0)

SD – standard deviation, x̄ – mean

*Presented as n (%) unless specified otherwise.
†Maternal sepsis was defined as fever + severe abdominal pain or smelly vaginal discharge. eclampsia/pre-eclampsia as severe headache + blurred vision or puffy face or convulsions or high blood pressure, maternal anaemia as severe anaemia or pallor and shortness of breath + being too weak to get out of bed or fast or difficult breathing, and maternal haemorrhage as any vaginal bleeding before labour.

Table S2. Women's characteristics for 842 maternal deaths*

**Age at death in years**	
12–15	5 (0.6)
16–19	83 (9.9)
20–29	415 (49.3)
30–39	287 (34.1)
40–49	52 (6.2)
**Highest education level**	
None	594 (70.5)
Primary	94 (11.2)
Secondary	103 (12.2)
Above secondary	51 (6.1)
**Highest education grade, $\bar{x}$ (SD)**	8.4 (3.4)
**What was her economic activity status in the year before her death?**	
Unemployed	16 (1.9)
Employed	19 (2.3)
Homemaker	804 (95.5)
Student	3 (0.4)
**Marital status**	
Married	836 (99.6)
Divorced	2 (0.2)
Widowed	1 (0.1)
**Stayed in the current house during fatal illness**	
Yes	773 (91.8)
No	65 (7.7)
Do not know	4 (0.5)
**The deceased stay at that time at:**	
Her own home at that time (different from the current location)	14 (22)
Her in-law's home	32 (49)
Her parent's home	12 (18)
Her brother's home	1 (2)
Do not know	6 (9)
**Time in years since living in a community where illness begin**	
<1	13 (1.6)
1–10	276 (33.2)
11–20	195 (23.4)
21–30	126 (15.1)
>30	151 (18.1)
Do not know	71 (8.5)
**Time in hours it took to reach nearest health facility**	
<1	436 (52.4)

1–2	305 (36.7)
>2–3	33 (4.0)
>3–4	9 (1.1)
>4	14 (1.7)
Do not know	35 (4.2)
**Place of death**	
Health facility	490 (58.2)
Home	222 (26.4)
On route to a health provider or facility	105 (12.5)
Other	25 (3.0)
**Signs and symptoms**	
Symptoms of the deceased during the illness that led to death	
Severe headache	206 (24.5)
Look pale (thinning/lack of blood) or have pale palms, eyes, or nail bed	191 (22.7)
Difficulty breathing	188 (22.3)
Fever	185 (22.0)
Bleed from anywhere	174 (20.7)
Unconscious	172 (20.4)
Vomit	171 (20.3)
Breathlessness	143 (17.0)
Abdominal pain	135 (16.0)
Fast breathing	95 (11.3)
Mental confusion	91 (10.8)
Convulsions	88 (10.5)
Chest pain	71 (8.4)
Cough	63 (7.5)
Urine problems	35 (4.2)
Severely thin or wasted	33 (3.9)
Painful neck	26 (3.1)
More frequent loose or liquid stools	25 (3.0)
Wheezing	21 (2.5)
Skin flakes off in patches	19 (2.3)
Blood in the liquid stools	18 (2.1)
Shingles or herpes zoster	14 (1.7)
Any lumps or lesions in the mouth	11 (1.3)
Any swelling or lump in the breast	10 (1.2)
Skin rash	10 (1.2)
Sores or ulcers	8 (1.0)
Ulcer (pit) on the foot	6 (0.7)
Ulcers (pits) in the breast	4 (0.5)
**Care-seeking for the fatal illness**	
Deceased received any care for fatal illness (first action)	
Sought no care	311 (36.9)
Home care	21 (2.5)
Traditional care/ pharmacist	14 (1.7)
Healthcare provider	496 (58.9)
Treatments received at home or health care provider during the fatal illness	517 (61.4)
Injectable antibiotics	271 (52.4)
Intravenous fluids	248 (48.0)

Blood transfusion	182 (35.2)
Have an operation for the illness	108 (20.9)
Nasogastric tube	85 (16.4)
Oral rehydration salts	51 (9.9)
Antiretroviral therapy	20 (3.9)
No treatment received	95 (18.4)
Conditions diagnosed by health professional recently or during the final illness	517 (61.4)
High blood pressure	146 (28.2)
Heart disease	51 (9.9)
Depression	32 (6.2)
Kidney disease	31 (6.0)
Liver disease	26 (5.0)
Asthma	19 (3.7)
Cancer	14 (2.7)
Diabetes	13 (2.5)
Chronic obstructive pulmonary disease	13 (2.5)
Dementia	10 (1.9)
Tuberculosis	8 (1.5)
Sickle cell disease	5 (1.0)
Dengue fever	4 (0.8)
Stroke	4 (0.8)
Epilepsy	3 (0.6)
**Social capital questions**	
**Activity/activities community participated together in the prior 12 months**	
One group identified	57 (6.8)
Two or more groups identified	36 (4.3)
No groups identified	749 (89.0)
**Respondent active in a community group in the prior 12 months**	
One group identified	16 (1.9)
Two or more groups identified	17 (2.0)
No groups identified	809 (96.1)
**Respondent able to turn to anyone for help during child's fatal illness**	
One person/group identified	21 (2.5)
Two or more persons/groups identified	40 (4.8)
No person/group identified	781 (92.8)
**HIV/AIDS questions**	
Ever have a positive HIV test	8 (1.0)
Any diagnosis by a health professional that the deceased had AIDS	0 (0.0)
The deceased's spouse/partner ever had a positive HIV test	8 (1.0)
Was there any diagnosis by a health professional that the deceased's spouse/partner had AIDS	2 (0.2)
**Delays for stillbirths and maternal deaths**	
Delay in recognising the severity of illness and danger signs (n = 845)	709 (83.9)
Delay in decision-making (n = 845)	302 (35.7)
No male member/any member available to accompany	19 (2.2)

No available person to take care of the children, the home, and the livestock	2 (0.2)
Fear of being ill-treated in the health facility	22 (2.6)
Lack of encouragement from relatives and community members to seek care does not c	231 (27.3)
Lack of money to pay for medical expenses and cost of transportation	126 (14.9)
Too sick to travel	22 (2.6)
Fear of long waiting time	10 (1.2)
Delay due to transport (n = 845)	151 (17.9)
Distance from a woman's home to a facility or provider	110 (13.0)
Lack of roads or poor condition of roads	56 (6.6)
Lack of emergency transportation	114 (13.5)
Delay in getting appropriate service from the provider (n = 845)	563 (66.6)
Lack of health care personnel care provider	22 (2.6)
Gender-related issues between patients and health care providers	0 (0.0)
Shortages of supplies, i.e. emergency medicines or blood	72 (8.5)
Lack of competence of health care providers to provide emergency care	526 (62.2)
Delayed referral	310 (36.7)
Long waiting time	2 (0.2)

SD – standard deviation, $\bar{x}$ – mean

*Presented as n (%) unless specified otherwise.
